# Self-supervised learning for remote sensing scene classification under the few shot scenario

**DOI:** 10.1038/s41598-022-27313-5

**Published:** 2023-01-09

**Authors:** Najd Alosaimi, Haikel Alhichri, Yakoub Bazi, Belgacem Ben Youssef, Naif Alajlan

**Affiliations:** Department of Computer Engineering, College of Computer and Information Sciences, King Saud University, 2454, Riyadh, 11451 Saudi Arabia

**Keywords:** Computational science, Computer science, Software

## Abstract

Scene classification is a crucial research problem in remote sensing (RS) that has attracted many researchers recently. It has many challenges due to multiple issues, such as: the complexity of remote sensing scenes, the classes overlapping (as a scene may contain objects that belong to foreign classes), and the difficulty of gaining sufficient labeled scenes. Deep learning (DL) solutions and in particular convolutional neural networks (CNN) are now state-of-the-art solution in RS scene classification; however, CNN models need huge amounts of annotated data, which can be costly and time-consuming. On the other hand, it is relatively easy to acquire large amounts of unlabeled images. Recently, Self-Supervised Learning (SSL) is proposed as a method that can learn from unlabeled images, potentially reducing the need for labeling. In this work, we propose a deep SSL method, called RS-FewShotSSL, for RS scene classification under the few shot scenario when we only have a few (less than 20) labeled scenes per class. Under this scenario, typical DL solutions that fine-tune CNN models, pre-trained on the ImageNet dataset, fail dramatically. In the SSL paradigm, a DL model is pre-trained from scratch during the pretext task using the large amounts of unlabeled scenes. Then, during the main or the so-called downstream task, the model is fine-tuned on the labeled scenes. Our proposed RS-FewShotSSL solution is composed of an online network and a target network both using the EfficientNet-B3 CNN model as a feature encoder backbone. During the pretext task, RS-FewShotSSL learns discriminative features from the unlabeled images using cross-view contrastive learning. Different views are generated from each image using geometric transformations and passed to the online and target networks. Then, the whole model is optimized by minimizing the cross-view distance between the online and target networks. To address the problem of limited computation resources available to us, our proposed method uses a novel DL architecture that can be trained using both high-resolution and low-resolution images. During the pretext task, RS-FewShotSSL is trained using low-resolution images, thereby, allowing for larger batch sizes which significantly boosts the performance of the proposed pipeline on the task of RS classification. In the downstream task, the target network is discarded, and the online network is fine-tuned using the few labeled shots or scenes. Here, we use smaller batches of both high-resolution and low-resolution images. This architecture allows RS-FewshotSSL to benefit from both large batch sizes and full image sizes, thereby learning from the large amounts of unlabeled data in an effective way. We tested RS-FewShotSSL on three RS public datasets, and it demonstrated a significant improvement compared to other state-of-the-art methods such as: SimCLR, MoCo, BYOL and IDSSL.

## Introduction

With the new generations of sensors, the complexity of remote sensing scenes has increased significantly due to its Very High Resolution (VHR)^1–3^, which poses a big challenge for RS scene classification. In recent years, researchers have developed many solutions for RS scene classification^4–7^, and deep Convolutional Neural Networks (CNN) have demonstrated strong capabilities in the field^8^. However, CNN models require huge labeled datasets for effective learning, and unfortunately, the availability of labeled data is still a big challenge in RS. So far, all RS datasets are small in size (thousands or tens of thousands of images), while in the computer vision area, for example, datasets like ImageNet^9^ have more than 14 million images, all of which are labeled. That is why, herein, a standard solution is to use a CNN model, pre-trained on ImageNet and then fine-tuned on our own target RS dataset, which is known as the transfer learning approach^8,10,11^. In this approach, the knowledge acquired by the pre-trained model is transferred to the ImageNet domain via fine-tuning the model on the target RS dataset. However, the transfer learning approach still needs large amounts of the target RS dataset to be labeled in order to get an accuracy value that is close to 100%. Otherwise, it would still fail when there are a limited number of labeled images, known as the few shot scenario. This is due to the large domain difference between the source domain (ImageNet) and the target RS domain, especially for multispectral or hyperspectral data, which are quite different from RGB data. On the other hand, it is relatively easy to acquire large amounts of unlabeled images in the RS field. Thus, methods that can learn potentially useful knowledge from large amounts of unlabeled images are highly beneficial. Recently, a novel idea known as Self-Supervised Learning (SSL) has appeared in the computer vision field that can help us achieve that^12^. It has been effectively applied in other fields such as computer vision^13–15^, natural language processing^16–19^, and object detection^20–22^.

**Figure 1:**
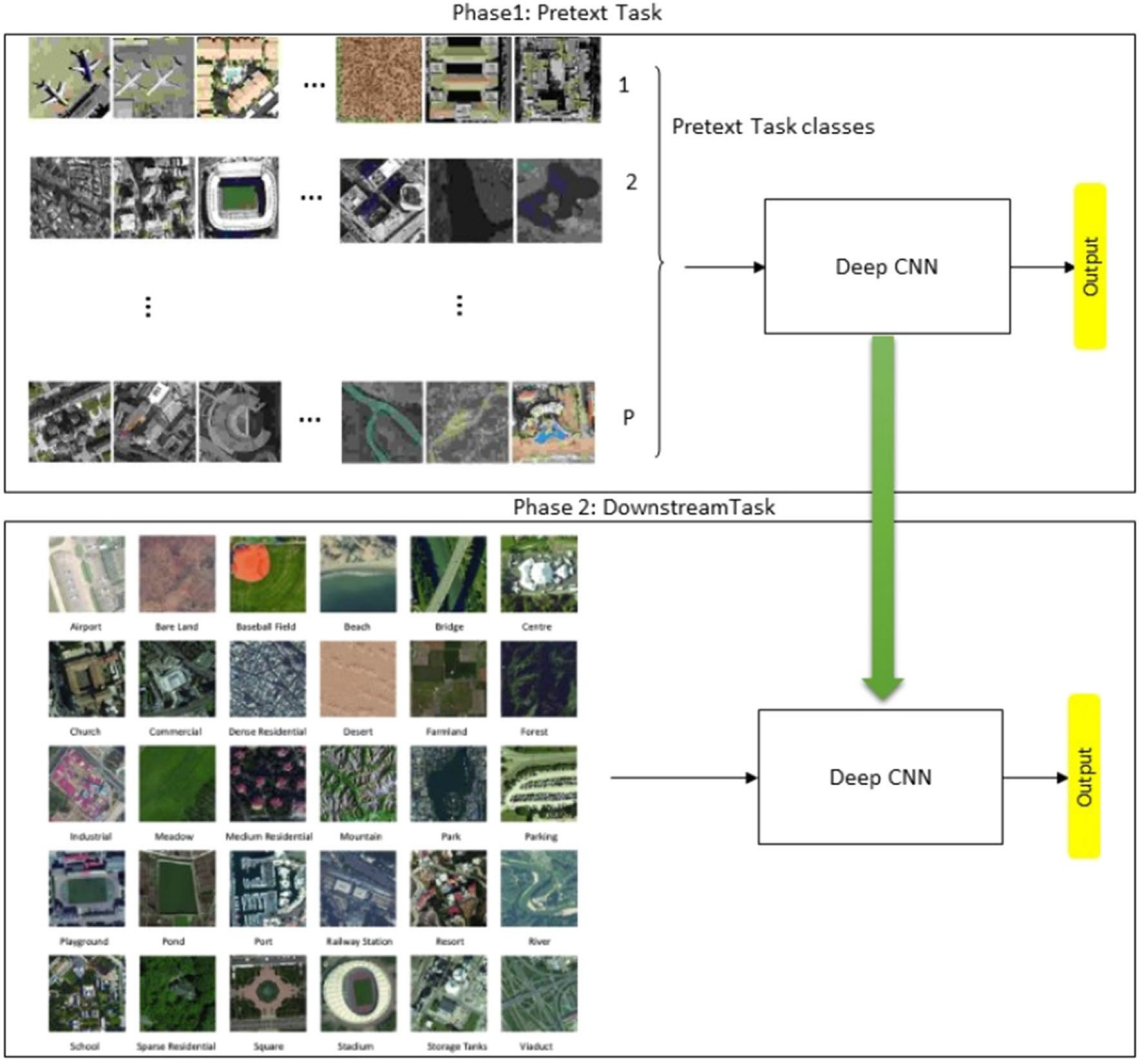
Illustration of the SSL framework, which consists of two phases of training. In phase 1, the model is pre-trained on the pretext task which uses pseudo labels assigned to the images. In phase 2, the model is fine-tuned on the downstream task using the labeled data.

SSL methods can learn discriminative feature representation from large amounts of unlabeled images by solving a pre-designed task (called the pretext task), and then transfer them to target task, known as the downstream task. Pretext tasks are designed so that a network trained to solve them will learn visual features that can be easily adapted to the downstream task^23^. The SSL paradigm is illustrated in Fig. 1. In the first stage, we pre-train the CNN model on a pretext classification task that uses all available unlabeled images in the target RS dataset. At the end of this first stage, the CNN model should be able to extract discriminate features from the target RS images. Then, in the second stage, the knowledge learned from the pretext task is transferred to the main downstream classification task using transfer learning^24^.

In order to learn the representation, some SSL methods define a pretext task as an auxiliary handcrafted classification task^23^. For example, the pseudo labels in the pretext task can be rotation angles, where all images are rotated at 0, 90, 180 and 270 degrees, respectively. In this pretext task, all images with the same rotated angle are considered to belong to the same class, and thus, all unlabeled images now have a pseudo label according to one of the four rotation classes. When a CNN model is pre-trained on this pretext task (classification into one of the four rotation classes), we anticipate that it will learn how to extract descriptive features from the target RS dataset such that when we move to the second stage, the CNN model can transfer this knowledge to the downstream classification task and provide good performance.

A more successful pretext task is based on the cross-view prediction framework^23^. In this approach, the model learns representations by predicting different views of the same image from one another. The views here are generated from each image using geometric transformations, such as rotation, scale, flipping, cropping, and so on. However, this approach has been shown to suffer from the model collapse problem, which happens when a model learns the same exact representation for all image. In that case the prediction problem becomes trivial. Recently, a more successful SSL method known as the cross-view contrastive pretext task has been proposed that has achieved very good performance in self-supervised learning^25–27^. Methods like the Simple Framework for Contrastive Learning of Visual Representations (SimCLR)^25^ and Momentum Contrast (MoCo)^26^ change the learning problem from a prediction of one view from another view to a discrimination between views that is generated from the same image as well as the views generated from a different image. Augmented views of the same image constitute a positive class, while augmented views of other images constitute a negative class. During the pretext task, a model is trained to optimize a loss function that maximizes the similarity between views for the positive class while minimizing the similarity between the features of the positive and negative classes. Cross-view contrastive methods often require comparing each example with many other examples to work well, and thus, they are quite slow in practice due to the negative samples that need to be considered during training, which causes training images to be sampled as negative pairs more than once during each epoch^28^.

One recent contrastive SSL method, known as Bootstrap Your Own Latent (BYOL)^28^, avoids the use of negative samples. Instead, BYOL minimizes the distance between different transformation views or augmentations of each sample image. BYOL is composed of an online CNN network and a target CNN network that are copies of each other. The main idea of BYOL consists of two different image views being passed to the online and the target CNNs, and then each CNN extracts a feature vector from its corresponding view and a predictor module is used to predict the target feature vector from the online feature vector. The whole architecture is optimized by minimizing the loss between the target feature vector and the prediction made by the online model. In order to avoid the model collapse problem, contrastive methods, such as BYOL^28^, use very large batch sizes during training, which requires high computational resources. However, these hefty computation resources, may not be available all the time.

In this paper, we propose an SSL solution for the classification of remote sensing images under the few shot scenario where there are only few (less than 20) labeled samples per class. The method is named RS-FewShotSSL and is pre-trained on a cross-view contrastive learning pretext task which tries to match different augmentation views of the same RS image. The downstream task is the classification of RS images with a limited number of labeled samples. RS-FewShotSSL has an architecture similar to BYOL^28^, with online and target networks used during the pretext task. The online network is composed of a feature encoder, projector, and predictor, while the target network has only an encoder and a projector which are copies from the online network. However, our proposed method uses an EfficientNet-B3 CNN^29^ as a feature encoder in both the online and target branches. We also use larger fully connected layer sizes in the projection and prediction modules and a more efficient activation function called the Swish function^30^. To address the problem of the limited computation resources available to us, we down-sample the images during the pretext task to 64 × 64 × 3 and use a batch size of 256. However, in the downstream task, using the down-sampled images results in highly-degraded accuracies. Subsampling the images reduces it higher frequency details. Higher frequency details are like local features, while low frequency details are like global features. Higher resolution images have higher frequency detail which enables the convolutional layers in the encoder to extract localized feature details. The encoder works better by learning to extract both local and global features. Thus, we propose a different architecture for the downstream task, where our model has two branches both having a copy of the EfficientNet-B3 CNN pre-trained during the pretext task. One branch uses down-sampled images of size (64 × 64 × 3), while the other branch uses the images with their original size (256 × 256 × 3). This architecture allows us to reduce the need for high computational resources while benefiting from both large batch sizes and full image sizes. Our contributions via this paper can be summarized in the following points:

- We develop a method for the classification of RS scenes under the few shot scenario, named RS-FewShotSSL, which can learn from the abundant available unlabeled RS scenes using self-supervised learning techniques.
- The architecture of the RS-FewShotSSL method uses the recent EfficientNet CNN as a backbone model, benefitting from its strong feature extraction capabilities.
- In the downstream task, we built a two-branch fusion architecture, with one branch using large batches of down sampled images and the other using small batches of full-size images. This architecture allowed us to reduce the memory requirements of the model while enhancing its classification accuracy.

The remainder of the paper is organized as follows. Section “Related work” provides an overview of related work, while in Section “Methodology”, we present our approach for self-supervised classification. Then, Section “Experimental results” presents the experimental results that show the capabilities of our method on common RS scene datasets, and finally, we give conclusions and future research directions in Section “Conclusions”.

## Related work

### SSL methods for RS scene classification

A few recent SSL methods have been proposed for the RS scene classification problem. In one of the earliest works, Yao et al.^31^ proposed a local manifold-constrained self-paced deep learning SSL method. First, the model is trained on the labeled samples (scenes). Second, the trained model is used to predict pseudo labels for all unlabeled samples. Then, the novel idea is to iteratively select simple trustworthy samples, append them to the training set, and retrain the model. The method uses a constraint to ensure the consistency of the pseudo-labels of the selected samples with the local manifold of labeled samples. This will increase the trustworthiness or confidence in the selected samples and their pseudo-labels. They tested their method on the NWPU-RESISC45 data set.

Another early method, by Yang et al.^32^, combines spatial pyramid matching using sparse coding (ScSPM) with self-supervised learning. ScSPM is a three-stage algorithm, composed of dictionary learning, sparse representation, and classification. In their method, Yang et al. proposed to perform the dictionary learning stage using SSL on the unlabeled samples. They also proposed to use a much larger auxiliary dataset (namely the Caltech-101 data set) to perform SSL in the dictionary learning stage. Then, they use the learned dictionary to classify RS datasets. So, in a sense they are also performing knowledge transfer from one dataset to another. Again, this method does not deal with the problem of few labeled shots.

In other work, Zhao et al.^24^ proposed a multitask learning framework to enhance both the ability to extract features and to generalize the CNN models. This framework combines the tasks of SSL and scene classification with dynamic weighting by adopting a mix-up loss strategy, and also enables the CNN to learn discriminative features without increasing the number of parameters. However, this work does not deal with the few labeled shots problem, it only tries to improve typical classification with CNN models with a large amount of labeled samples. In^33^, Guo et al. proposed a method for RS scene classification, called self-supervised gated self-attention generative adversarial networks (GANs). They introduce a gated self-attention module was introduced into GANs to concentrate on key scene areas and remove unavailing information. They also introduced a pyramidal convolution block into the residual block of the discriminator to take various levels of details in the image. These two points are meant to reinforce feature representation of the model. The only relation to SSL in their work is that they included the unlabeled scenes in their training by adding a similarity loss component in the discriminator loss function. Again, they do not address the few shot problem in their work. In^34^, Stojnic et al. study the Contrastive Multiview Coding (CMC) method^35^ for self-supervised pre-training application of SSL. They analyze the influence of the number and domain of images used for self-supervised pre-training on the performance on downstream tasks. They confirm that, for the downstream task of RS image classification, using self-supervised pre-training on RS images can give better results than using supervised pre-training on images of natural scenes.

More recently, Tao et al.^36^ proposed a method similar to ours, called Instance Discrimination Self-Supervised Learning (IDSSL). In fact, they compare three types of pretext tasks: (1) Image inpainting, (2) Predict the relative position, and (3) Instance discrimination (or what is mostly known as cross-view contrastive learning) for RS scene classification and find that instance discrimination is more effective. Then, they present a method using this SSL approach under the few shot scenario (5, 10 and 20 labeled samples). The back bone CNN used in their work is ResNet50^37^. The results of this study encouraged us to adopt of SSL in remote-sensing scene classification^36^.

### Relation to semi-supervised learning and few shot learning (FSL) paradigm

SSL can learn potentially useful knowledge from unlabeled data, thus eliminating the need for costly labeling efforts^12^. In that sense, SSL is similar to semi-supervised learning, which also learns from unlabeled data; however, the way each method uses it, is different. In semi-supervised learning, we optimize the structure of the unlabeled data through a specific loss function. For example, we can use entropy as a loss function in order to make the unlabeled data more uniformly distributed across classes^38^, because, in general, image classification datasets are balanced (meaning that there are almost the same number of images/scenes in each class). Thus, ensuring a uniform distribution across classes preserves the structure of the data and helps in reducing misclassification errors.

In contrast, SSL defines a pretext task in which the model learns image representations from unlabeled data through assigning handcrafted pseudo labels for the images, predicting image views from one another, or discriminating between views of the same image versus views from other images.

In our work, we also assume that there are few labeled images per class in the target dataset, which is similar to few shot learning (FSL)^39^. FSL is an important research direction in RS and machine learning in general; however, the FSL paradigm is slightly different from the SSL paradigm in that FSL aims to recognize classes unseen during training given only a few labeled samples, whereas in SSL methods there is no assumption that the model did not see the classes before. In fact, it is quite the opposite; in SSL methods, the model is pre-trained on all unlabeled images representing all classes of the dataset. In other words, the model is exposed to all classes during pre-training, albeit without knowing which image belongs to which class.

## Methodology

We describe the proposed RS-FewShotSSL solution in this section. First, we introduce EfficientNet-B3 CNN model as the backbone CNN model used as a feature encoder. Then, we discuss the details of our proposed RS-FewShotSSL method.

### EfficientNet convolutional neural networks (CNN)

EfficientNet is one of the most effective CNN models in the literature. It was proposed by Tan et al.^29^ and has shown impressive results in many image classification tasks^40,41^. Tan et al. used an automatic search algorithm to optimize the base architecture of EfficientNet, which they called EfficientNet-B0. Then, they uniformly scaled all the dimensions of depth, width and resolution using a compound coefficient with a set of fixed scaling coefficients to generate seven more larger models^29^. Using these techniques, they were able to develop a family of eight CNN models, called EfficientNet-B0 to EfficientNet-B7, with impressive performance on the ImageNet^9^ computer vision dataset compared to previous state-of-the-art CNN models^29^. This family of EfficientNet models is ordered in terms of number of parameters, where EfficientNet-B0 is the smallest model with about 4 million parameters, and EfficientNet-B7 is the largest, with more than 66 million parameters. In this study, we implemented the pre-trained EfficentNet-B3 model, which consists of more than 12 million Parameters.

### Proposed self-supervised RS-FewShotSSL method

**Figure 2:**
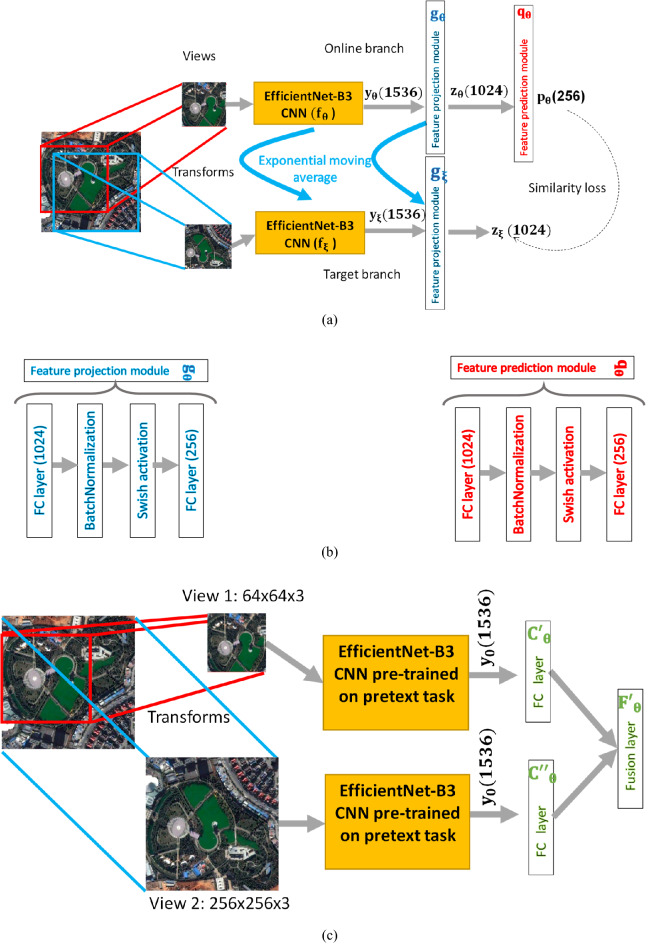
Architecture of the RS-FewShotSSL method for RS scene classification. (**a**) the architecture used for the pretext task, (**b**) the details of the projection and prediction layers, and (**c**) the architecture used for the downstream task.

Previous SSL methods based on contrastive learning need large batch sizes to work. It turns out that computing the similarity loss function between the features of the online and target networks using a large batch of images is indirectly forcing the model to contrast between positive and negative samples within the batch. We observed this behavior when we experimented with the use of small batch sizes during pretext training. In this case, the performance of the model in the downstream task is severely downgraded. In fact, in the original papers on SimCLR^25^ and BYOL^28^, the batch size used was 4096, distributed over four Graphical Processing Units (GPU). This was important to speed up the training on the huge ImageNet dataset. However, due to our limited computational resources, there is a memory constraint on the batch size. Given an image size of 256 × 256 × 3, the maximum batch size possible is 32. We deal with this problem by using small scale image views with a size of 64 × 64 × 3 during pretext training, which allows for large batches of size 256. However, the classification performance of the model is quite limited in this case. Our proposed solution, RS-FewShotSSL, is motivated by this dilemma and is shown in Fig. 2.

#### Pretext task

Figure 2a shows the architecture used in the pretext task. The solution is composed of an online and a target branches that both employ the EfficientNet-B3 CNN model as a feature encoder. The online branch consists of three modules: an encoder ${f}_{\theta }$, a projector ${g}_{\theta }$, and a predictor ${q}_{\theta }$, as illustrated in Fig. 2a, while the target branch has only an encoder ${f}_{\xi }$, and a projector ${g}_{\xi }$. The target branch is a copy of the online branch minus the predictor module, except that it has its own network weights. The projection and prediction modules make the two branches asymmetric which further helps the architecture avoid the representation collapse problem. The online network predicts the output of the target network using the prediction module. By training this model, the aim is to maximize the similarity between the prediction of the online network and the output of the target network.

During training, the weights of the online network are updated normally through the backpropagation algorithm. This is in contrast to the target network, which is updated via an exponential moving average of the online network’s weights. The target network provides the regression targets to train the online network, and its parameters $\xi$ are an exponential moving average of the online parameters $\theta$. More precisely, given a target decay rate $\tau\, in\, [0, 1]$ after each training step, we perform the following update:

1 $$\xi \to \tau \xi +\left(1-\tau \right)\Theta .$$

Given a set of images $D$, an image $x \sim D$ sampled uniformly from $D$, and two distributions of image augmentations $\tau$ and $\mathop \tau \limits{^{\prime}}$, the method starts by generating two augmented views from $X$ by augmenting the image representations $t \sim \tau$ and $t \sim \mathop \tau \limits{^{\prime}}$. Each branch will produce an output ${y}_{\theta }$ and a normalized projection ${z}_{\theta }$. Then, the predictor generates a normalized prediction ${p}_{\theta }$ from the online network. Again, the loss function is based on the cosine similarity of Eq. (2), and is defined as follows:

2 $${\mathcal{L}}_{\theta ,\xi }\triangleq 2-2 . {S}_{cosine} \left\{ {p}_{\theta } , {z}_{\xi }\right\} =2-2 \frac{{ {p}_{\theta } }^{T} . {z}_{\xi } }{\Vert {p}_{\theta }\Vert \Vert {z}_{\xi }\Vert }.$$

The cosine similarity is in the range {− 1, 1} with 1 indicating high similarity and − 1 indicating dissimilarity. Thus, the way the loss function ${\mathcal{L}}_{\theta ,\xi }$ is defined makes it positive and inversely proportional to the similarity. It follows that the online branch is updated by the regression loss function as the regression targets are given by the target network.

The details of the projection and prediction layers are shown in Fig. 2b. We set the hidden layer size to 1024 and the output layer size to 256. In addition, we also used the swish activation function instead of the standard ReLU. The Swish activation function was introduced by Howard et al.^30^ in their design for the MobileNet CNN model, and is defined as follows:

3 $$Swish\left(x\right)= x . \sigma \left(\beta x\right)= \frac{x}{1+ {e}^{-\beta x}} ,$$

where $\sigma \left(\beta x\right)$ is the sigmoid function and $\beta$ is a trainable parameter. When $\beta =1$, this is known as the sigmoid-weighted linear unit function. Finally, we emphasize that the architecture of the pretext task is trained with small-scale images with a size of 64 × 64 × 3 pixels. This allowed us to use large batches with a size of 256.

#### Downstream task

Then, during the downstream task, we discard the target network and only kept the feature encoder ${f}_{\theta }$ of the online network, which is now able to produce self-learned representations for all samples in the training data. The learned representations are self-consistent; they are invariant under different transformations of the data^28^. Then, a new classification layer is appended to the encoder and the whole model can be fine-tuned on the downstream task. This is similar to what is usually done during fine-tuning of pre-trained CNN models. Indeed, the pretext step simply produces a pre-trained model that can be fine-tuned using typical transfer learning techniques.

Figure 2b shows our proposed architecture for the downstream task. It is composed of a two-branch fusion architecture with each branch consisting of a copy of the CNN feature encoder ${f}_{\theta }$. Then, we append two output layers, called C’ and C’’, which are basically fully connected layers with a Softmax activation function that provide output classification probabilities. Finally, the fusion layer computes the mean probabilities of the two output layers.

The CNN feature encoder ${f}_{\theta }$ of both branches are initialized by the weights from the pretext stage. However, during fine-tuning on the downstream task, the upper model takes in as input image views of size 64 × 64 × 3, while the lower one can take image views with the original size of 256 × 256 × 3. This clever approach provided a good compromise between resource costs and performance.

#### Views generation via augmentation

**Figure 3:**
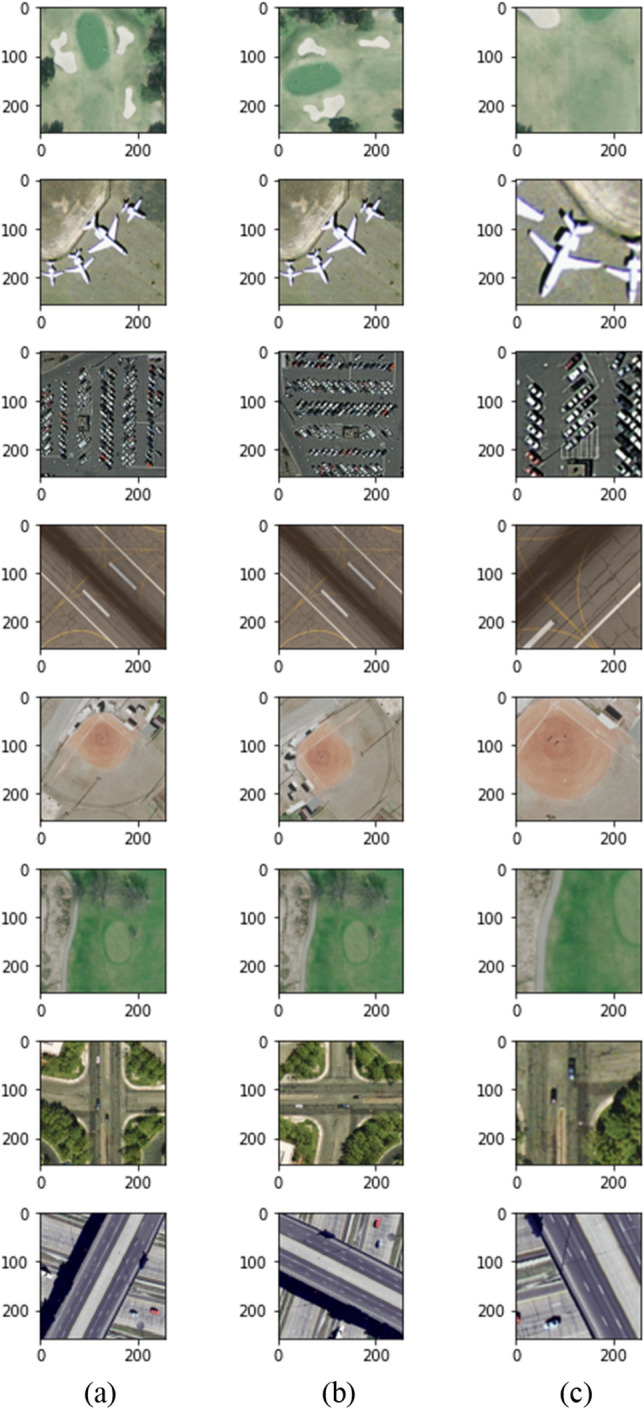
Sample augmentation views generated during training. (**a**) original images in a batch, (**b**) random views for online CNN, and (**c**) random views for target CNN.

To generate the augmentation views, we used the following geometric transformation: random crop and resize, random flip, and random rotation by 90°. However, unlike previous work, we used different transformation parameters, including color jitter with a strength of 0.4 and a probability of 0.8, a minimum cropping size of 20% of the original size, a random gray scale with a probability of 0.2, and finally, vertical flip, horizontal flip, and random rotation all with probabilities of 0.5. We did not use Gaussian blur because we had already down-sampled the images from 256 × 256 × 3 to 64 × 64 × 3, and adding Gaussian blur, will significantly reduce the quality of the images and potentially cause severe degradation. Figure 3 exhibits an example of a batch of images and the random views generated for both the online and target CNN encoders.

#### RS-FewShotSSL algorithm

The following algorithm shows the main steps of the proposed method. In particular, the datasets are split into training, unlabeled, and test sets. We follow the same method for splitting the data as in the literature (see^36^ for example) in order to be able to compare our work.
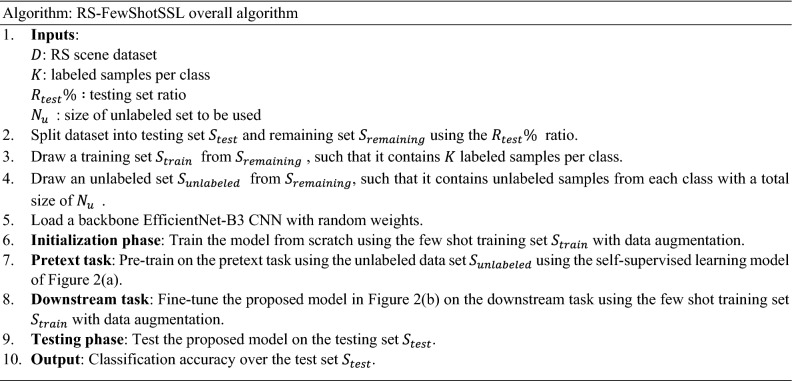


## Experimental results

In this section, we present the experimental work of this study. First, we describe the datasets used, then we explain how we set up and executed the experiments, and finally we present the results and compare them to state-of-the-art methods.

### Dataset description

To evaluate the scene classification model in remote sensing, we used three datasets called UCMerced^42^, AID^4^, and NWPU-RESISC45^1^. Table 1 provides a summary of the different properties of these datasets.

**Table 1:** Details of datasets of RS scenes used in this work.

	Dataset name:		
	UCMerced	AID	NWPU
No. of images	2100	10,000	31,500
No. of classes	21	30	45
Images/class	100	Varies	700
Image size	256 × 256	256 × 256	256 × 256
Resolution	0.3 m/pixel	0.5–0.8 m/pixel	0.2–30 m/pixel

The UCMerced dataset, published in 2010, is one of the earliest datasets, and it has only 2,100 images distributed over 21 classes. The size of this dataset makes it unsuitable for deep learning models, which need large amounts of data; however, the amount of data augmentation used in the proposed methods should compensate for this shortcoming. In addition, it can be beneficial for model analysis and fine-tuning because of its low processing time requirements.

These other two datasets, AID and NWPU-RESISC45, or NWPU for short, are large and diverse. The AID is a dataset acquired ftom different remote imaging sensors in different countries around the world^4^. It is a collection of 10,000 annotated aerial images distributed in 30 land-use scene classes, and can be used for image-classification purposes. Further, the NWPU-RESISC45 dataset was created by Northwestern Polytechnical University (NWPU)^1^, and includes 31,500 images separated into 45 scene classes, with 700 images in each class.

### Experiment setup and training protocol

We implemented the proposed deep SSL learning solution using PyTorch^43^, which is a deep learning library written in Python. We executed the various experiments on a Lambda server with intel® Xeon(R) Silver 4214 CPU running at a clock frequency of 2.20 GHz housing 48 CPU’s, 128 GB of RAM, and two NVIDIA TITAN RTX GPUs with 25 GB of memory each.

For the pretext task, all scenes are resized to 64 × 64 × 3 and with that we are able to set the batch size to 256. Then, we optimize the model in Fig. 2a for 400 epochs using the LARS optimizer^44^, with a cosine decay learning rate schedule^45^ without restarts. We set the base learning rate to 0.2, scaled linearly^46^. We also used a warm-up period of 10 epochs where the learning rate is constant at 0.001. In addition, we use a global weight decay parameter of 1.5 × 10^−6^ while excluding the biases and batch normalization parameters from both the LARS adaptation and weight decay.

For the main downstream task, we fine-tune the model in Fig. 2b for a total of 60 epochs, with a batch size of 32, and using the Adam optimizer^47^ with a learning rate of 0.0001, which is reduced at epoch 30 to 0.00001.

For performance evaluation, we present the results using the overall accuracy (OA), which is the ratio of the number of correctly classified samples to the total number of the tested samples. OA is the standard way to evaluate RS scene classification methods, and is claimed in^48^ to be the most suitable. It is defined by the following formula:

4 $$OA= \frac{\sum_{i=1}^{C}{n}_{ii}}{\left|{S}_{test}\right|} ,$$

where ${n}_{ii}$ is the number of correct classifications for class $i$ in the test set, $C$ is the number classes, and $\left|{S}_{test}\right|$ is the total number of test samples. Below, we present the full algorithm for the RS-FewShotSSL method.

### Results and comparison to state-of-the-Art SSL methods

In this section, we evaluate the performance of RS-FewShotSSL method for the three RS datasets. For each dataset we tested two few shot scenarios. For the AID and NWPU-RESISC45 datasets, we used five and 20 labeled samples per class. However, UCMerced is a small dataset with only 100 samples per class. Thus, using 20 samples is equivalent to 20% of the data used as labeled data, which is not consistent with other datasets where the labeled data is below 6% of the dataset. Thus, for UCMerced, we do not use 20 labeled samples per class, instead, we use three and five samples per class as shown in Tables 2 and 3.

**Table 2:** Classification accuracy values obtained using UCMerced dataset as the batch size is varied from 256 down to 8.

	UCMerced	
	Samples/class	
	3(3%)	5(5%)
ImageNet Pre-trained	70.05 ± 1.74	80.00 ± 0.78
**Batch size during pretext task**		
256	62.62	79.57
128	61.67	73.57
64	56.67	70.95
32	54.52	69.52
16	55.95	66.93
8	52.14	65.02

**Table 3:** RS-FewShotSSL results for the UCMerced dataset and comparison to state-of-the-art methods.

Methods:	UCMerced	
	Samples/class	
	3(3%)	5(5%)
ImageNet Pre-trained	70.05 ± 1.74	80.00 ± 0.78
MoCo v2^49^, 2020	60.95	80.00
SimCLR^25^, 2020	65.71	79.76
BYOL^28^, 2020	56.43	81.43
RS-FewShotSSL no-multiscale [ours]	62.62	79.57
RS-FewShotSSL [ours]	**74.76 ± 078**	**83.57 ± 0.88**

Significant values are in bold.

In the first experiment, shown in Table 2, we test the proposed method on the UCMerced dataset to illustrate the importance of using large batch sizes for self-supervised learning. For this experiment, we fix the image size to 64 × 64 × 3, which allows us to experiment with batch sizes of up to 256 for both the pretext and downstream tasks.

As we mentioned before, in the downstream task, the target network is discarded and only the online network is fine-tuned on the labeled samples. We fixed all the other parameters of the method as described in the experimental setup section. Then, we varied the batch size starting from 256 down to 8 and reported the OA of the downstream task. As can be seen from Table 2, the OA in the downstream task degrades as we decrease the batch size, confirming the importance of using large batch sizes for SSL methods based on cross-view contrastive learning.

Next, we present the in Tables 3, 4, and 5 the final results of the RS-FewShotSSL method, for the UCMerced, AID, and NWPU-RESISC45 datasets, respectively. To show the effectiveness of RS-FewShotSSL, we compared it to many state-of-the art SSL methods including: (1) SimCLR^25^, (2) MoCo v2^49^, (3) BYOL^28^, and (4) IDSSL^36^. We obtained the results for the methods MoCo v2, SimCLR, and BYOL by ourselves, but the results for the MARTA GANS and IDSSL methods were reported by the authors of^36^. We note here that, usually, we aim to run each experiment at least five times and report the mean and standard deviations for the performance for all methods. However, due to time constraints, especially since each method takes a few hours to complete, we opted to execute the experiments for the methods MoCo v2, SimCLR, and BYOL methods only once. This explains why we do not report standard deviations for these methods. However, for our proposed method, we do execute the experiments 5 times using a different random labeled set for every time and report the mean and standard deviation of the OA result.

**Table 4:** RS-FewShotSSL results for the AID dataset and comparison to state-of-the-art methods.

Methods:	AID	
	Samples/class	
	5 (1.5%)	20 (6%)
ImageNet Pre-trained	75.42 ± 1.24	88.40 ± 0.73
MARTA GANS^50^ 2017	53.08 ± 0.44	61.90 ± 0.60
MoCo v2^49^ 2020	57.02	65.36
SimCLR^25^ 2020	60.40	74.50
BYOL^28^ 2020	62.45	77.19
IDSSL^36^ 2022	76.80 ± 0.30	80.62 ± 0.22
RS-FewShotSSL [ours]	**83.27 ± 1.03**	**87.13 ± 0.67**

Significant values are in bold.

**Table 5:** RS-FewShotSSL results for the NWPU-RESISC45 and comparison to state-of-the-art methods.

Methods:	NWPU-RESISC45	
	Samples/class	
	5(0.73%)	20(2.95%)
ImageNet Pre-trained	65.97 ± 1.55	83.44 ± 1.34
MARTA GANS^50^ (2017)	43.52 ± 0.18	59.01 ± 0.24
MoCo v2^49^ 2020	56.71	63.15
SimCLR^25^ 2020	61.28	70.69
BYOL^28^ 2020	64.97	78.03
IDSSL^36^ 2022	**80.62 ± 0.03**	85.80 ± 0.15
RS-FewShotSSL [ours]	73.37 ± 1.38	**88.03 ± 0.43**

Significant values are in bold.

First, we observe that the obtained results are better than the ones yielded by fine-tuning an EfficientNet-B3 model that is pre-trained on the ImageNet^9^ dataset (denoted by a pre-trained ImageNet in the first row of each table). This clearly shows that RS-FewShotSSL is able to learn from the large amounts of unlabeled data and enhance its classification accuracy.

The second observation is that when using five labeled samples per class, the OA results improved and outperformed the other SSL methods discussed in the literature, except for NWPU-RESISC45. In this scenario, the IDSSL method by Tao et al.^36^ has achieved 80.62 ± 0.03, while our method obtained 71.18 ± 0.88. Nevertheless, upon inspection, we found that Tao et al.^36^ had obtained those results by using all of the unlabeled samples available, which totaled 25,200, whereas we have only used 10,000 unlabeled samples that we selected randomly from the 25,200. We did that to reduce the running time of the experiments due to the time limitations for access to the computational platform. Tao et al.^36^ have also obtained results from using 10,000 unlabeled samples and achieved an overall accuracy value of only 68.52, which is lower than our result of 71.18 ± 0.88.

The other observation is that most of the time, the results of the proposed RS-FewShotSSL method are improved by simply fine-tuning an EfficientNet–B3 model pre-trained on the ImageNet dataset. The only exception we found is for the AID dataset with 20 labeled samples per class; in this case, fine-tuning the ImageNet pre-trained model achieved 88.40 ± 0.73, while the proposed RS-FewShotSSL method only achieved 87.13 ± 0.67. We also observed that the improvement was more significant for the larger AID and NWPU datasets compared to the UCMerced, which is due to the large size of the unlabeled set in AID and NWPU. Recall that the size of the unlabeled set used were 1680, 8000, and 10,000 for UCMerced, AID, and NWPU respectively, and since deep learning models benefit greatly from much larger datasets, the improvement provided by the proposed RS-FewShotSSL over the ImageNet pre-trained models was more significant for the AID and NWPU datasets.

**Figure 4:**
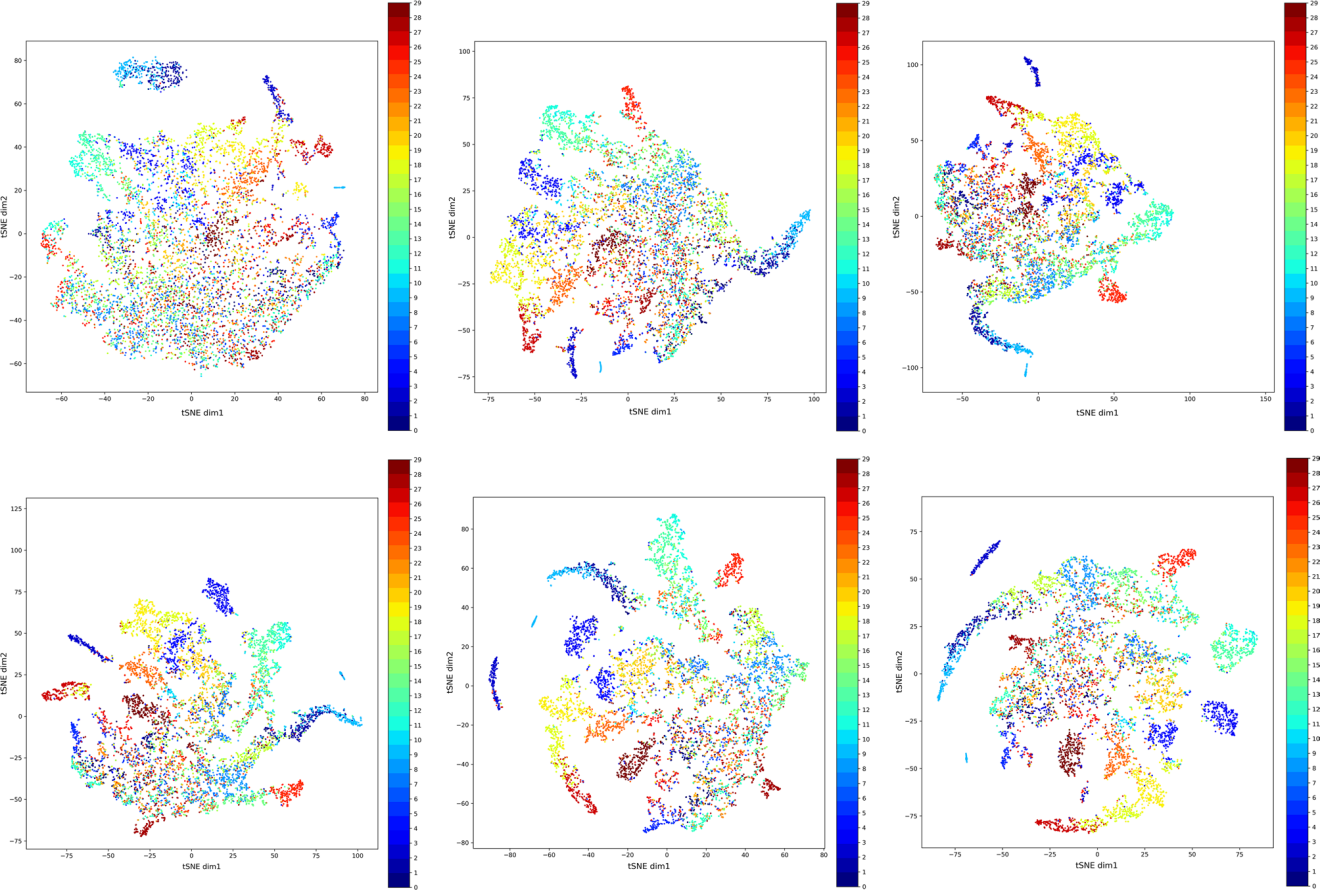
tSNE plots for the unlabeled set of the AID dataset throughout the training process. The number of epochs is 300 and plots are saved every 50th epoch.

To illustrate the effectiveness of the self-supervised learning in the proposed method, we used the Distributed Stochastic Neighbor Embedding (t-SNE)^51^ technique to visualize the distributions of the feature vectors extracted by the backbone CNN model. t-SNE is a non-linear technique used for data exploration and visualizing high-dimensional data. We show in Fig. 4 tSNE plots of the distribution of feature vectors extracted from the unlabeled set during a sample pretext training phase. We generated these plots every 50th epoch, so the figure shows plots for epochs 50, 100, 150, 200, 250 and 300. As one can see, the unlabeled data start with the classes mixed together. Then, over the training, the model slowly learns to extract features that cluster together based on the image classes. Of course, there is still some confusion between classes, but remember that these samples are unlabeled, and that the model is learning to cluster them nevertheless.

**Figure 5:**
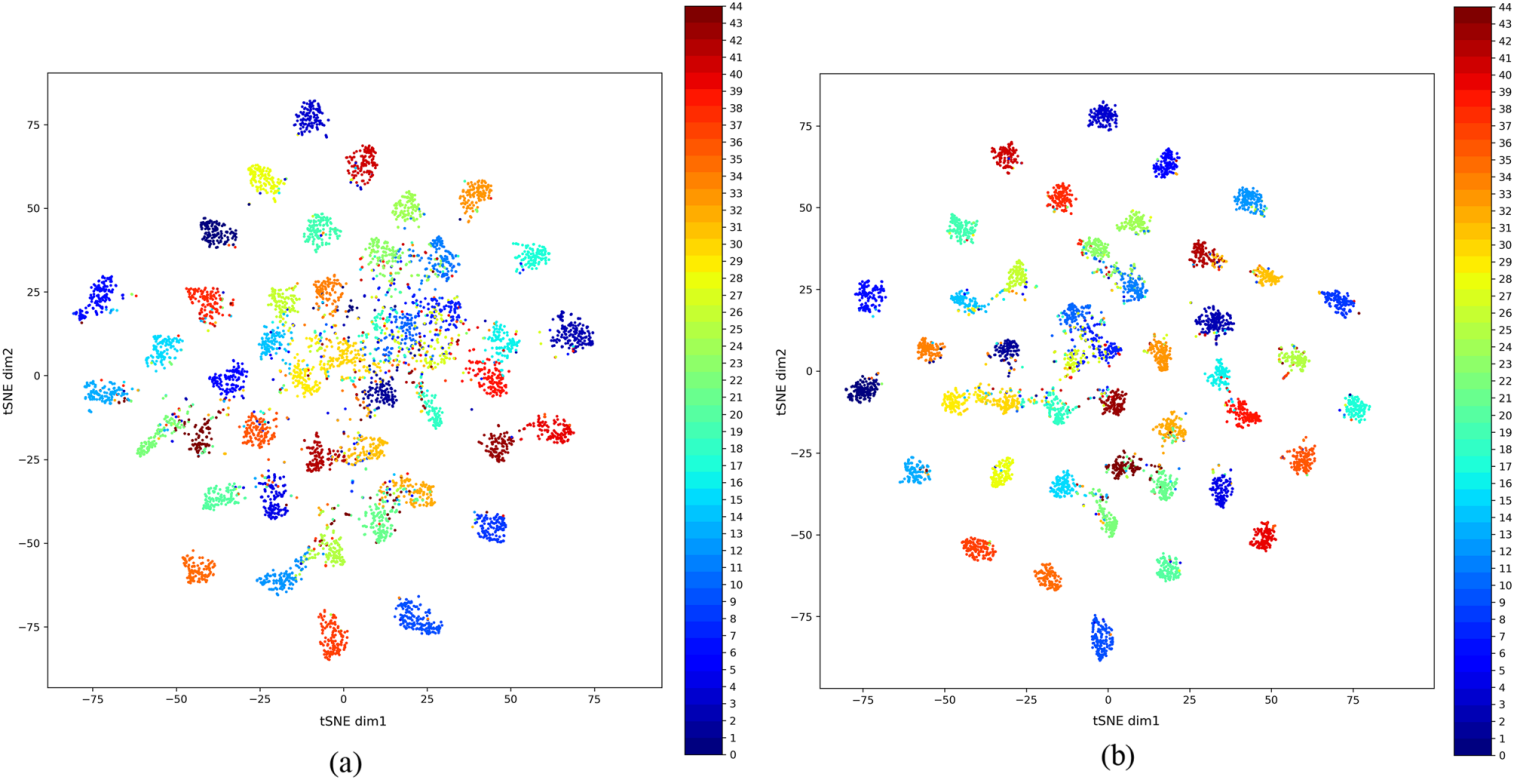
tSNE plots for the test set of the NWPU dataset. (**a**) plot after supervised training on the training set by fine-tuning the pre-trained efficientNet-B3 model, (**b**) plot after supervised training on the training set (downstream task) by fine-tuning the RS-FewShotSSL model pre-trained on the unlabeled set (pretext task).

Finally, in Fig. 5 we show the tSNE plots for the testing set of the NWPU dataset for the case of 20 samples per class. The plot in Fig. 5a shows the distribution of the testing set features produced by fine-tuning the pre-trained ImageNet model, while the plot in Fig. 5b shows the distribution of the testing set features produced by the proposed RS-FewShotSSL model. From the distribution plots, we can see that the proposed RS-FewShotSSL method is able to learn features that enable it to classify the images better. Thus, the proposed method is able to effectively utilize the unlabeled set to enhance the quality and discriminability of its learned features, which in turn enhances the final classification accuracy.

## Conclusions

In this study, we developed a new SSL paradigm for scene classification of remote sensing images under the few shot scenario, called RS-FewShotSSL. It is based on an enhanced cross-view contrastive SSL approach similar to BYOL combined with the EfficientNet-B3 CNN model. In this study, we have found that cross-view contrastive SSL methods that do not use negative samples, such as BYOL, are actually indirectly doing a contrastive loss between positive and negative samples, because of the use of the batch normalization layers on large batches of unlabeled data during the pretext training phase. Thus, in order for these methods to work, we need to use large batch sizes (BYOL used 256 to 4096). Our proposed method consists of a novel deep learning architecture that can be trained using both high-resolution and low-resolution images. Thereby, the latter allow for larger batch sizes which significantly boosts the performance of the proposed pipeline on the task of RS classification. We tested RS-FewShotSSL on three public datasets, namely USMerced, AID, and NWPU-RESISC45, and the results showed that RS-FewShotSSL outperforms the recent SSL methods for supervised classification downstream tasks under the few shot scenarios utilizing 5 and 20 labeled samples per class.

Future work should focus on overcoming the issue of large batch sizes. Solutions to this problem include using distributed computations or another, completely different SSL method. Another direction is expanding the model to learn from more unlabeled data from multiple datasets. This is a challenging problem due to the data distribution shift between the datasets, in addition to the fact that these datasets do not share the same image classes. Here, domain adaptation techniques should be considered and utilized.

## Data Availability

The three datasets of UCMerced, AID, and NWPU-RESISC45 used in this study are publicly available at: http://alhichri.36bit.com/research.html. The original dataset copies are located in the following URLs respectively: http://weegee.vision.ucmerced.edu/datasets/landuse.html. https://captain-whu.github.io/AID/. https://doi.org/10.6084/m9.figshare.19166525.v1.

## Code availability

Code used to run experiments in this paper is available here: https://github.com/alhichri/RS-FewShotSSL.
